# Retentive Characteristics of a New Attachment System for Hybrid Dentures

**DOI:** 10.3390/ma13153434

**Published:** 2020-08-04

**Authors:** Christin Arnold, Charlotte Stampa, Ramona Schweyen, Jeremias Hey, Arne Boeckler

**Affiliations:** Department of Prosthetic Dentistry, University School of Dental Medicine, Martin-Luther-University Halle-Wittenberg, Magdeburger Str. 16, 06112 Halle, Germany; charlotte.stampa@uk-halle.de (C.S.); ramona.schweyen@uk-halle.de (R.S.); jeremias.hey@uk-halle.de (J.H.); arne.boeckler@zahnzentrum-halle.de (A.B.)

**Keywords:** overdenture, Locator, Novaloc, abutment, retention force, implant angulation

## Abstract

Removable implant-anchored dentures have become an established treatment concept especially for older, multimorbid patients. This study investigates the retention force (RF) of two different attachment systems. A total of 96 specimens (n = 8 for each condition) were fabricated and RF was measured under different conditions: fatigue (10,000 cycles dislodging), thermal undulation (5/55 °C, 5000 cycles) and implant-angulation (0°, 5°, 10°, 15°, 20°). The Novaloc system ((N), 0° and 15° abutments, yellow matrix (Y)) was compared to the Locator system ((L), pink (P) and orange (O)). Initial RFs (8.57 ± 0.99 N (NY), 19.39 ± 8.10 N (LP), 8.8 ± 5.28 N (LO)) were reduced by ageing simulation (26% (NY), 66% (LP), 89% (LO); *p* < 0.001). After thermocycling, Novaloc’s RFs decreased by 33% (*p* < 0.001) while the Locators’ RFs increased by 34% (LP: *p* = 0.002, LO: *p* = 0.148). In contrast to LP, the RFs of Novaloc abutments and LOs predominantly showed no clinically relevant dependence on implant angulation. Ageing processes tended to result in lower RFs at higher implant angulation. Thus, the Novaloc attachment system offers an alternative to Locator attachments. It is characterized by a comparatively continuous RF-curve over the entire wearing period. Future clinical studies have to be conducted to verify the in vitro demonstrated advantages of the Novaloc system.

## 1. Introduction

In geriatric dentistry, the removable implant-anchored denture represents an established treatment concept. The insertion of two interforaminally positioned endosseous implants has become an established method for secure anchorage of a full denture [1,2,3,4], yielding improved denture function and patient satisfaction [5,6,7]. Moreover, implants offer the fundamental advantage preventing resorption of the alveolar bone [8,9]. In multimorbid patients, unsplinted attachment systems, such as o-rings or magnets, have been proven to be superior to splinted attachments, as bars, especially in terms of cleaning, handling, and stability and offer the advantage of adaptability to changes in the oral situation. These individual attachments are characterized by low space requirements, comparatively low acquisition costs, and simple application. Moreover, dental prostheses anchored on individual attachments can usually be repaired easily. Clinically, individual attachments such as spherical head anchors, magnets, and Locators or bars have proven to be standard solutions [10,11,12,13].

Depending on the jawbone content, deviations of the implant axes ranging from 0.5° to 27° in the horizontal and between 0.1° and 12.9° in the sagittal direction were found in everyday clinical practice [14,15]. Moreover, the insertion and removal direction of the prosthesis performed by the patients often does not correspond to the axial insertion direction of the connecting elements [14]. Unfortunately, individual attachments on heavily angulated implants show increased wear, often resulting in their frequent replacement [16,17]. For strong angulations, the ideal treatment is a bar restoration [14,17]. However, for strongly diverging or converging implants, individual attachments, such as the Locator system (ZestAncors, Escondido, CA, USA), are clinically used. In such cases, however, only a limited adjustment of the retention performance by selecting special matrices (Locator Replacement Males) is possible, and a primary angulation compensation at the level of the attachment cannot be not ensured. Despite yielding positive results for retention behavior in in vitro investigations [7,18], the Locator system shows increased wear behavior in the form of repair and maintenance susceptibility [19]. To address this issue, systems with angled abutments (patrices) are becoming increasingly available on the market. For example, the Novaloc Retentive System for hybrid dentures (Insitut Straumann AG, Basel, Switzerland) offers an abutment angled at 15° in addition to straight abutments. Moreover, the abrasion-resistant surfaces (amorphous diamond-like carbon (ADLC)) of these abutments are intended to have a positive influence on the wear behavior.

Wear and the consequent loss of retention is basically the most frequent prosthetic complication with all individual connecting elements. Since retention and wear behavior are crucial for patient satisfaction [20], many studies in the relevant literature have assessed the connecting elements. These retention forces vary considerably (0.2 to 20 N), not least due to different study designs [11,18,21,22,23,24,25,26]. Evidence-based data on the retention forces required for optimal chewing function in removable dentures are not available.

Thus, the objective of the present study was to compare the Novaloc and Locator attachment systems with respect to retention behavior before, during, and after simulated ageing at different implant angulations. Matrix inserts with approximately equal retention forces according to the manufacturer’s specifications were used. As a primary null hypothesis, it was assumed that for the selected matrices, the initially measured pull-off forces correspond to the manufacturer’s specifications, regardless of the implant angulation, and that the retention forces among the different abutment systems are approximately the same, according to the selection. The second null hypothesis assumed that no clinically relevant retention force changes occur due to ageing simulations on the attachment systems. As the third null hypothesis, it was assumed that with the Straumann Novaloc Retentive System, there is no difference between a 15° secondary part at an implant angulation of 15° (20°) and a 0° secondary part at 0° (5°) angulation.

## 2. Materials and Methods

A detailed list of the system-specific components used can be found in Table 1. The Novaloc test series included the standard secondary parts with and without angulation. Both abutments were combined with yellow matrices. To allow observation of retention forces at comparable implant angulations with reference to the Locator attachment system, the Locator attachment was combined with pink and orange Locator Replacement Males. On the basis of the manufacturers’ recommendations, tests were performed at different implant angulations (Table 1).

Sample size calculation was based on the results of Stephens et al., Mínguez-Tomás et al., and Elsyad et al. [17,27,28], with a power of 0.8 and a level of significance of 0.05. In total, 96 individual attachment combinations were produced for this study. Each test series (n = 12, Table 1) consisted of eight specimens each.

### 2.1. Specimen Fabrication

**Figure 1:**
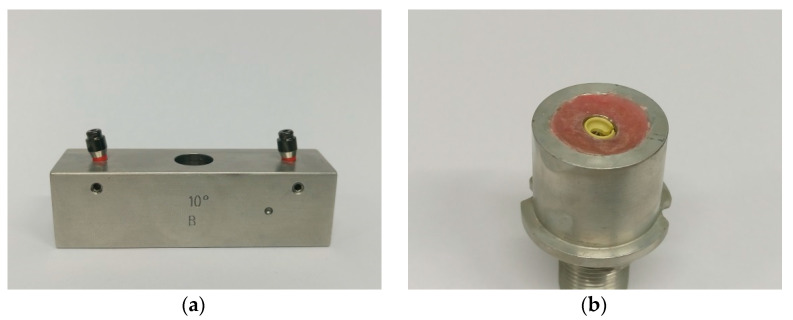
(**a**) Part 1: metal block—analog or abutment holder; (**b**) part 2: PMMA base material with matrix holder and matrix; example shown: implant angulation—10° with the Novaloc attachment system—0° and yellow matrix.

**Figure 2:**
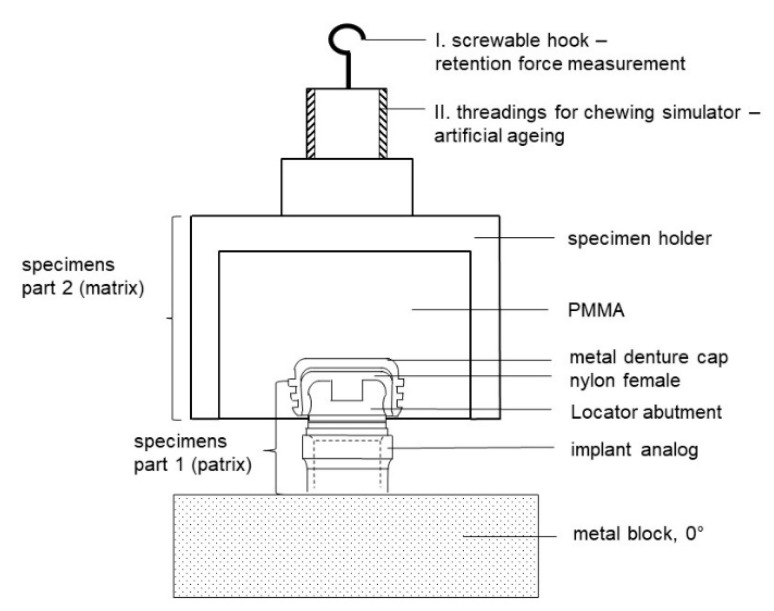
Schematic drawing: specimen attachment to the universal testing machine (I) and to the chewing simulator (II); example shown: implant angulation—0° with the Locator attachment system—0° and pink matrix.

A specimen basically consisted of two parts. The lower part of the specimen simulated the patient’s jaw. It contained the implant analog (RN analog; length, 12 mm; material, stainless steel; Institut Straumann AG, Basel, Switzerland) with the screw-retained abutments (N and L). Especially for this purpose, metal blocks of stainless steel (68 mm × 20 mm × 20 mm) were fabricated with exactly matching drill holes for all implant angulations to be tested (Figure 1a, showing an example of 10° implant angulation). Within the metal block, analogs were fixed by screws. The denture base was represented by the upper specimen portion (D 16 mm × H 20 mm, part 2) (Figure 1b). The matrix holder was embedded into this according to the manufacturer’s instructions for the lower part of the specimen (abutment). Finally, the respective matrix was integrated into the matrix holder (Figure 2). The base material for the fixation was autopolymerizing polymethyl methacrylate (PMMA; PalaXpress; Kulzer GmbH, Hanau, Germany). The fixation of the specimen holders (part 1 and part 2) could be performed reversibly both in the universal testing machine (Z010; Zwick Roell, Ulm, Germany) and in the chewing simulator (Willytec chewing simulator, CS-4.4; SD Mechatronics, Feldkirchen-Westerham, Germany) (Figure 2; I and II). The initial embedding of the matrices or matrix holders was carried out in the chewing simulator according to the manufacturer’s instructions.

### 2.2. Retention Force Measurement

All retention force measurements were obtained on the universal testing machine. Part 1 of the specimen was attached to the lower traverse, and part 2 of the sample was fixed manually onto the patrix. A hook that could be screwed into the axial course of the specimen holder (part 2, Figure 2; I) facilitated the connection to the upper traverse and the load cell via a loosely supported steel cable. Lateral forces could thus be excluded. To ensure that the weight of the specimens would not influence the retention forces, the force with the specimen (part 2) hanging on the steel cable was set to zero before the respective adaptations. In addition, the specimen components to be joined were wetted with artificial saliva (Glandosane; Cell Pharm, Bad Vilbel, Germany). At each measurement time point, 20 pull-offs were made per specimen combination at a pull-off speed of 50 mm/min. The matrix was completely detached from the patrix (pull-off path: 4 mm). Using the test software (testXpertII V2.2; Zwick Roell, Ulm, Germany), in addition to the maximum force in N, the mean value, standard deviation and variance, and the respective force-displacement diagrams were automatically displayed.

### 2.3. Artificial Ageing

All specimens were subjected to an ageing simulation after the initial pull-off forces were determined. For this purpose, 10,000 insertions and removals of a denture were simulated in the chewing simulator. After every 100, 200, 500, 1000, 5000 and 10,000 cycles, the specimens were removed and retention force measurements were taken on the universal testing machine. The simulation was performed in a saliva bath mixture (Aqua dest; Glandosane; ratio: 1:2). Finally, all specimens (part 2) were artificially aged in the thermocycler with 5000 alternating cycles between a hot (55 °C) and cold (5 °C) bath of distilled water. The immersion time was 30 s, and the dripping time was 17 s.

### 2.4. Release Period

**Figure 3:**
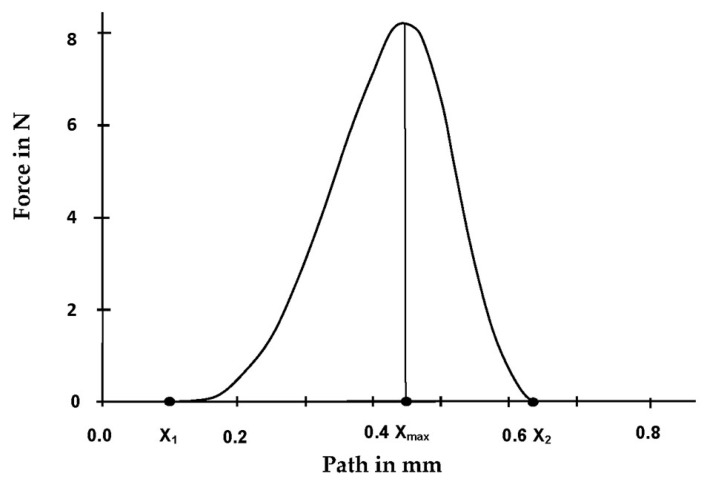
Graphical representation of a retention force measurement with an example force-displacement diagram: implant angulation—0° with a Locator attachment system—5° and a pink matrix.

To verify a statement about the release period and possible differences in the separation of the respective attachment systems, three force-displacement diagrams of each initial specimen combination from the initial pull-off tests were evaluated, as shown in Figure 3. The time values (*x*-axis) were taken and, using the formula proposed by Petropoulus et al. [10], the release periods in which the matrix detaches from the patrix after reaching the maximum retention force were calculated (1):

(1) $$\mathrm{Release}\;\mathrm{period}\;=\frac{\mathrm{Deflection}\;\mathrm{at}\;\mathrm{release}\;\left(X_{2}-X_{1}\right)-\mathrm{Deflection}\;\mathrm{at}\;\mathrm{maximum}\;\mathrm{force}\;\left(X_{\max}-X_{1}\right)}{\;50\;\mathrm{mm}/\min\left(\mathrm{Pull}-\mathrm{off}\;\mathrm{speed}\right)}$$

### 2.5. Microscopic Measurement

**Figure 4:**
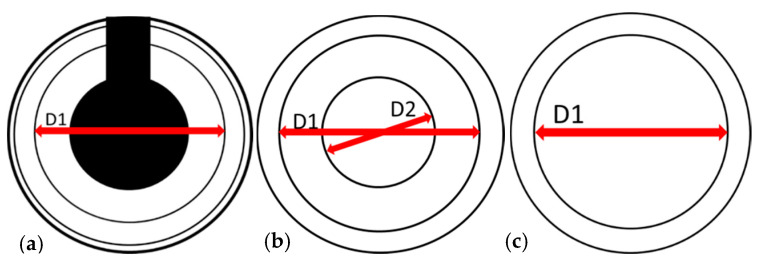
Light microscopic measurement of the diameters at the matrices. (**a**) Novaloc—yellow; (**b**) Locator—pink; (**c**) Locator—orange.

Apart from the material, the geometry of the abutments or matrices influences the retention force. Dimensional changes caused by wear can cause retention force changes. To verify these, all matrices were measured by light microscopy initially and after simulated ageing (measurement and inspection device: VMZM 40, software VIS/METRONA; -4H-JENA Engineering GmbH, Jena, Germany) in accordance with the illustrations (Figure 4).

### 2.6. Statistical Analyses

At all measurement time points, 20 pull-off values were created for each specimen; thus, the sample size per test series and measurement time point was n = 160. All data were listed in Excel, transferred to the SPSS program (IBM SPSS Statistics 25, International Business Machines Corporation, New York, NY, USA), and descriptively analyzed in terms of the following problems:

#### 2.6.1. Comparison of the Mean Value of the Initially Measured Retention Forces with the Manufacturer’s Specifications, Independent of the Implant Angulation

In accordance with the matrix color coding, the initially measured values were averaged and compared with the manufacturer’s specifications. Any deviations were stated as percentages.

#### 2.6.2. Comparison of Mean Values of the Initially Measured Retention Forces with the Retention Forces after the Ageing Simulations, Independent of the Implant Angulation

On the basis of the matrix color coding, the initial mean values were compared with the mean values after insertion and removal simulation and after thermal cycling. The Kolmogorov–Smirnov and Shapiro–Wilk tests were used to check the results of the individual test series for normal distribution. Levene’s test was used to check for homogeneous variances. The *t*-test for paired samples was used to check for significances.

#### 2.6.3. Comparison of the Mean Values of the Retention Forces of the Different Attachment Systems Measured Initially and after Ageing, Independent of the Implant Angulation

Since, according to the manufacturer’s specifications, matrices of medium retention forces were used in this study, the various test series were also compared with each other at the same measurement time points. Here, the *t*-test was applied to independent samples. For all comparisons, the effect sizes were calculated using values from the test statistics (*t*-tests) according to Dunlap et al. [29].

#### 2.6.4. Differentiation—Individual Test Series

Influence of artificial ageing according to matrix color coding and implant angulation

In order to avoid distorted *p*-values due to an extremely large sample size, the 20 pull-off values per sample and measurement time point were averaged for statistical comparisons. After testing for normal distribution and variance homogeneity (Kolmogorov–Smirnov, Levene’s test), single-factor analysis of variance (ANOVA) with repeated measurements was used to check for significances. In the absence of homogeneity of variances, Welch ANOVA with Dunett T3 post-hoc test was used.

#### 2.6.5. Influence of Implant Angulation on the Attachment Systems

Under the given conditions, the t-test (ANOVA) for independent samples was used to compare the mean retention force values within an attachment system at different angulations. This comparison of the retention forces was performed per angulation initially, after 10,000 insertions and removals, and after thermocycling.

#### 2.6.6. Comparison of Release Periods During Retention Force Measurements

For the comparisons, the release periods per attachment system were summarized on average. Due to missing preconditions (Kolmogorov–Smirnov, Levene’s test *p* ≤ 0.05), the comparison was carried out by means of the Mann–Whitney U test.

## 3. Results

### 3.1. Mean Value Comparison of the Initially Measured Retention Forces in Comparison to the Manufacturer’s Specifications, Independent of the Implant Angulation

The initial retention forces (mean value, standard deviation, and median) of the differently coded matrices are shown in Table 2, independent of the angulations, in comparison to the manufacturer’s specifications and the values obtained after artificial ageing and the thermocycling process.

The pull-off value of the yellow Novaloc matrix is specified as medium in the product description and defined at 12.01 N. On average, the yellow Novalocs initially achieved a 28.64% lower value, independent of the angulation. In contrast, the initially measured retention value of 19.39 N of the pink Locator matrices was 45.35% higher than the specified 13.34 N. On average, only the orange Locator matrices initially reached the specified manufacturer value.

### 3.2. Mean Value Comparison of the Initially Measured Retention Forces Compared to the Retention Forces after The Ageing Simulations, Independent of the Implant Angulation

**Figure 5:**
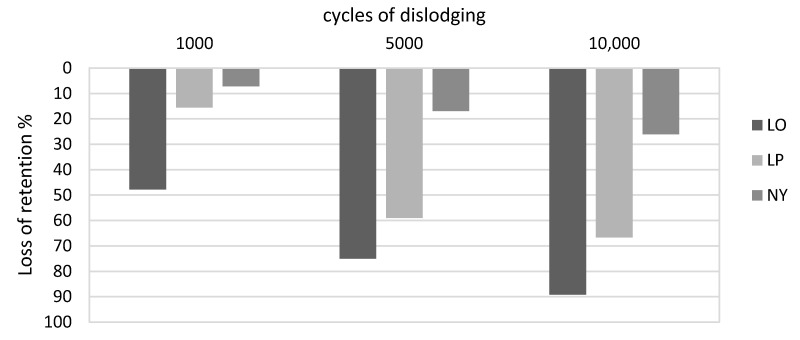
Percentage retention force loss compared to the initial RFs, independent of the (implant) angulations.

In comparison with the average initially measured retention force values (Figure 5), simulation of 10,000 insertions and removals resulted in an average retention force loss of 66% (*p* < 0.001) in the pink Locators and 89% (*p* < 0.001) in the orange matrices. For the Novaloc system, the retention force was reduced by an average of 26% (*p* < 0.001). For all three attachment systems, a strong effect is confirmed with d > 1 in the mean value comparisons.

Compared to the initial measurements for the pink (*p* = 0.002) and orange (*p* = 0.148) Locators, the thermocycling process produced an average retention force increase of 34%. On the Novalocs, on the other hand, the values decreased by 33% (*p* < 0.001) compared to the initial values. The significances of these differences (*p* ≤ 0.05) were confirmed by a strong effect size (d > 0.9).

### 3.3. Mean Value Comparison of the Retention Forces of the Different Attachment Systems Measured Initially and after Ageing, Independent of the Implant Angulation

Mutual comparisons of the independent test series show that, on average, only the initial values of the Locator system with the orange matrix inserts did not differ significantly from the Novaloc system with yellow matrix inserts (*p* = 0.824). After 10,000 cycles, there was no difference in retention force between the pink Locator system and the Novaloc system (*p* = 0.227). All remaining comparisons as well as the average retention forces after thermocycling showed differences with clinical relevance (*p* < 0.001).

### 3.4. Differentiation—Individual Test Series

#### 3.4.1. Influence of Artificial Ageing According to Matrix Color Coding and Implant Angulation

**Figure 6:**
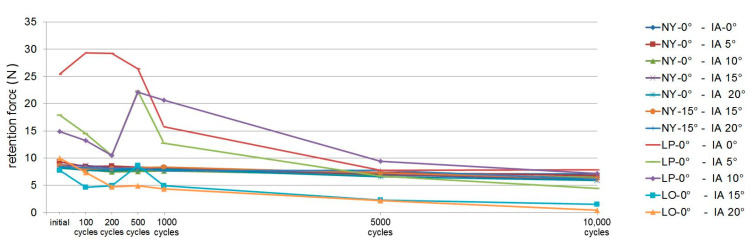
Retention force curves corresponding to artificial ageing per test series; for labeling, see Table 1.

The retention force curves of the individual test series are shown graphically in Figure 6. All test series were subject to wear reactions and predominantly showed retention force losses during the insertion and removal simulations. Only the Locator attachment system, with up to 1000 insertions and removals, showed a partial increase in the retention force. The mean values of the eight samples of each measurement series per measurement time point and the *p*-values ≤ 0.05 within the test series resulting from the statistical evaluation of the pairwise comparisons are listed in detail in Table 3.

#### 3.4.2. Influence of Implant Angulation on the Attachment Systems

**Figure 7:**
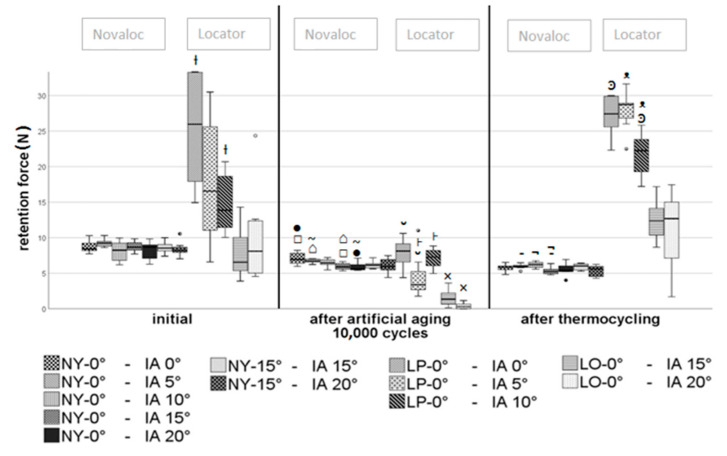
Median values as a function of implant angulation; for labeling, refer to Table 1. Significances: □ *p* = 0.006, ● *p* = 0.007, ⌂ *p* = 0.002, ~ *p* = 0.004; - *p* = 0.015, ¬ *p* = 0.003; × *p* = 0.031; Ɨ *p* = 0.006; _˘_ *p* = 0.018, ˫ *p* = 0.039, ͽ *p* = 0.008, _ᵜ_ *p* = 0.001.

In the statistical evaluation, the mean values of the initial retention force measurements after 10,000 insertions and removals and after thermocycling were considered as a function of the respective abutment systems and implant angulations:

On the straight Novaloc abutments, no significant differences were initially verified with various angulations (*p* ≥ 0.05). Some of them showed slightly lower retention forces after the ageing process of 10,000 cycles in specimen combinations with higher implant angulations (Figure 7). This could also be observed in individual cases after thermocycling. The retention forces of the angled Novaloc abutments did not show significant differences with respect to implant angulation at any measurement time point (*p* ≥ 0.05).

The orange Locator matrix inserts showed no significant differences initially and after thermocycling (*p* ≥ 0.05). Only after 10,000 insertion and removal cycles, a slightly (~1.13 N) smaller average retention force (*p* = 0.031) was determined at an implant angulation of 20°.

As shown in the box plot (Figure 7), the highest differences were found in the Locator system with pink matrix inserts. The highest mean retention forces were measured on axially positioned implants. As the implant angulation increased, the mean retention forces decreased (Figure 7). Due to the high standard deviation, only the comparison between the 0° and 10° implant angulations showed a statistically significant difference (10.5 N). The reduction in retention forces with increasing implant angulation was also reflected as a tendency after the ageing processes. After 10,000 insertions and removals, retention forces differing by an average of 3 N were statistically determined in the comparisons between 0° and 5° and between 5° and 10° implant angulations (Figure 7). After thermocycling, there was no statistical difference between 0° and 5° implant angulation. However, a comparison of these two angulations with the 10° angulation showed 6–7 N lower retention forces.

Comparisons of the Novaloc combinations (NY-15°/IA-15° to NY-0°/IA-0° and NY-15°/IA-20° to NY-0°/IA-5°) with theoretically identical positioning of the Novaloc abutments relative to the matrix or denture pull-off direction showed no significant differences (*p* ≥ 0.05). The only exception was the comparison of NY-15°/IA-15° to NY-0°/IA-0° with 10,000 insertions and removals (~1 N, *p* = 0.020).

### 3.5. Comparison of Release Periods During Retention Force Measurements

Table 4 shows that, with the exception of NY-0°/IA-5°, the Novaloc-0° abutments had the shortest release periods (mean value: 0.00353 min, *p* < 0.001). The longest release periods were found in the Locator system with the pink matrix inserts (mean value: 0.00480 min, *p* < 0.001). Between these groups, the mean value of the Locator system with the orange matrices was 0.00368 min and that of the angled Novaloc abutment was 0.00397 min. Statistically, the differences between NY-0° and LO-0° (*p* = 0.658) and between NY-15° and LO-0° (*p* = 0.065) could not be confirmed.

### 3.6. Dimensional Behavior of the Matrices

**Figure 8:**
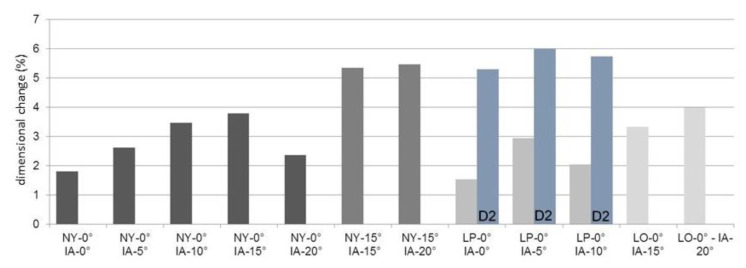
Percentage deviations of matrix geometry D1 (or D2) before and after simulated ageing; for labeling, refer to Table 1 and Figure 4.

As shown in Figure 8, there was a change in dimensions in all matrices. All cases showed an increase in the measured diameters (MWT D1: NY-0°: +100 µm, NY-15°: +193 µm, LP-0°: +78 µm, MWT D2: +112 µm, LO-0°: +132 µm). The change tended to increase with the implant angulation (Figure 8). Exceptions are NY-0°, –IA-20°, and LP-0°–IA-10°.

## 4. Discussion

In general, wear is characterized by mechanically and partially chemically induced surface material loss [30]. Wear-induced surface changes on color-coded matrices or abutments can cause changes in the retention force [31]. The results of this study can be compared with previous studies only to a limited extent due to the widely differing test setups and test parameters in the literature. Moreover, the individual habits of the patients cannot be simulated sufficiently. For retention force measurement, 20 pull-offs were made at a speed of 50 mm/min [10,32,33]. This speed is similar to the clinical situation when removing dentures [34]. Based on the assumption that patients remove removable dentures three times a day for cleaning, 10,000 insertions and removals were performed, representing a wearing period of about nine years [35].

### 4.1. Mean Value Comparison of the Initially Measured Retention Forces Compared to the Manufacturer’s Specifications Independent of Implant Angulation—Null Hypothesis 1

The null hypothesis that the pull-off forces, according to the matrix color coding, correspond to the manufacturers’ specifications had to be partially rejected. As shown in Table 2, only the orange Locator matrices corresponded to the manufacturer’s specifications in terms of mean values. The pink Locator matrices showed clinically relevant higher initial values, and the yellow Novaloc matrices showed clinically relevant lower initial values. The deviation of initial retention forces from the manufacturer’s specifications has been confirmed in the relevant literature. In most cases, increased retention forces, sometimes strongly increased forces, have been verified [11,18,31,36,37,38]. Besides a frequently visible initial break-in phase [18,37,38], the lack of information on test conditions, equipment, and pull-off speed on the part of the manufacturers is assumed to be the cause of these variations [37]. Depending on the test temperature, the specific coefficient of thermal expansion (CTE) of the matrix materials can also influence the retention forces in different ways by changing the dimensions of the matrices accordingly. In general, classic nylon materials can have twice the CTE of polyetheretherketone (PEEK). The investigations in this study were performed at a constant room temperature of 20 °C ± 2 °C. Exact CTE data from the manufacturers are not available. This problem could be a reason for the extremely low retention forces, which were quite apparent for the Novaloc matrices consisting of PEEK, in comparison with the manufacturer’s specifications. Dimensional variations within and between different matrix batches (production units) on the part of the manufacturers are also considered to be a cause of the deviating Locator retention values [27]. For a simulation close to reality, all pull-offs were performed with artificial saliva. In the literature, saliva applications led to a reduction in retention force on Locator attachments [39,40]. Explicitly for the Locator attachment system, retention forces were initially significantly higher. In the interest of the patients and to avoid overloading the implants, it therefore seems clinically advisable to perform the primary denture fixation at first with inserts of low retention specifications.

### 4.2. Mean Value Comparison of the Initially Measured Retention Forces Compared to the Retention Forces after the Ageing Simulations Independent of Implant Angulation—Null Hypothesis 2

#### 4.2.1. Ageing: Insertions and Removals

Both the average retention forces (LP: 66%, LO: 89%, NY: 26%) and the retention force curves of the individual test series basically showed a continuous, statistically significant loss of retention force from 1000 insertion and removal cycles onwards. Consequently, the second null hypothesis also had to be rejected. However, in the opinion of the authors, a retention force loss of 26% is not clinically relevant, or is relevant only to a limited extent. Moreover, in the literature, the reported relative retention force losses after artificial ageing range from 21% to 78.62% for Locator attachment systems [13,18,28,36,37,38]. Numerous test apparatuses are based on two implants [10,11,37]. Even though we considered individual attachments in this study, we could not exclude the wear-promoting transverse forces in the Locator test series during the joining of the specimen components due to the material properties of the matrices and the movable fixation of the nylon inserts. The stress caused by the swivel-joint connection [7] and the consequent deformation of the matrices may have produced an increased effect on the retention force. In contrast, the geometry of the Novaloc matrices and the PEEK structure allowed clear fixation of the upper specimen components along the abutment axis. Tilting movements and additional wear-promoting processes were thus greatly minimized. Therefore, the results of the Novaloc retention force measurements showed a smaller spread and frequently repeated retention force values, yielding more uniform force curves (Table 3, Figure 6). In vivo investigations also confirm the wear susceptibility of the Locator attachment system [15,41]. Apart from case studies [42], there are currently no clinical studies available for the Novaloc system. In the event of a clinically relevant retention force loss, the color-coded matrices of both systems can be replaced chairside with the aid of an instrument at a comparatively low cost and without great effort. The present test results suggest that such replacements will be required more often with the Locator attachment system (specifically Locator orange, see Table 3) compared to the Novaloc attachment system, or will not be necessary with the latter.

Besides the angulation of implant abutments, varying designs of matrices were presented in the last years to compensate implant divergence. Thus, future studies might evaluate the retentive behavior of angulated abutments of the Novaloc system in comparison to abutments with another construction principle, for example the OT Equator system (Rhein 83, Bologna, Italy). The Equator system in combination with the smart-box abutment system allows due to a tilting mechanism with a rotation fulcrum the passive insertion up to 50° implant angulation. Both in vitro and in vivo studies found this system to reveal acceptable results [43,44].

#### 4.2.2. Ageing by Thermocycling

In assessments based on the mean values, thermocycling produced a significant and clinically relevant increase in retention force, specifically for the Locators, in both the initial and in the retention force values after artificial ageing. For the Novalocs, on the other hand, the initial values and the values after artificial ageing decreased (Table 2). These values clearly show the strong dependence of the Locator on the surrounding environment. The manufacturer has not provided precise information on the composition of the nylon used. The classic nylon is polyamide 6.6 and is produced from hexamethylene diamine and adipic acid. In principle, compact polyamides have a high wear resistance and good sliding properties, but they can easily be attacked by acids and oxidizing chemicals. These can be mostly excluded during thermocycling, wherein only the oxidation process (passivation) of the sample holders made of aluminum caused by the alternating bath could have influenced the microstructure of the matrices toward higher retention forces. Polyamides are essentially linear polymers with regularly repeating amide bonds along the main chain. The amide groups interact with each other via hydrogen bonds and can be hydrolytically split again. Apart from the crystalline structure, the properties of the polyamides depend particularly on their water content. Polyamides react to the moisture content of the environment with reversible water absorption or release. The water is stored in the amorphous areas of the polyamide. This was also confirmed by subsequent measurements using an analytical balance. A three-hour storage of 15 pink Locator matrices in distilled water resulted in an average weight increase of 9% relative to the average initial weight. As confirmed by microscopic measurements, this leads to an increase in volume in the Locator matrices (specifically D2, Figure 8). This dimensional change leads to a reduction of the space reaching into the patrix and could therefore be the cause of a strong increase in retention force after thermocycling.

By adding additives (fibers etc.), the mechanical properties, or possibly the retention force, can be improved or changed. Consequently, however, the sensitivity to hydrolysis would increase due to the formation of macroscopically or microscopically small gaps between the matrix and additive. Liquids can be absorbed into the resulting spaces by capillary action. However, the manufacturer did not provide a definitive statement regarding the composition; thus, there is no information on changes to any new batches. Clinical applications often show heavy wear of the Locators [15,42], which could have been possibly compensated with further additives by the manufacturers. Both of these factors could be the reason for the fact that in other studies, less water absorption and thus not such a strong increase in retention force was observed [18].

### 4.3. Influence of Implant Angulation on the Attachment Systems—In Combination—Null Hypothesis 3

#### 4.3.1. Novaloc

On the Novaloc attachment system, the implant angulation initially showed no influence on the retention forces. The differences, which were partly statistically quantified after the ageing simulation (*p* ≤ 0.05), were not clinically relevant (see Figure 7).

#### 4.3.2. Locator

The same findings apply to the orange Locator inserts. When using the pink Locator inserts, a decrease in retention force was observed with increasing implant angulation, with partial clinical relevance. Yang et al. [16] and partly Stephensen [17] also noted this in their studies. The Locator patrix has a cylindrical shape with both internal and external undercuts. The matrix engages in these outer and inner undercuts. In contrast to the straight alignment, in which the retention is composed of the resistance of all undercuts, this relationship is disturbed by implant angulation, due to which the pull-off direction is not parallel to the axis of the abutment. The side facing the implant angulation has a reinforced undercut effect and the opposite side has a weakened undercut effect, which, in total, can lead to a reduction in the retention force [17].

In further studies on two implants with implant convergence and divergence, different retention force changes were verified [17,40]. In this respect, further studies explicitly assessing these factors in the Novaloc system are necessary.

The third null hypothesis, that there is no difference between a 15° secondary part at an implant angulation of 15° (20°) and a 0° secondary part at 0° (5°) angulation on the Novaloc attachment system, could be confirmed. The statistically quantified (*p* ≤ 0.05) difference after 10,000 insertions and removals was nearly 1 N, which is not clinically relevant. The retention force is ensured by the angle compensation of the Novaloc abutment, such that the same conditions exist with regard to undercuts and the pull-off direction.

### 4.4. Microscopic Measurement

The microscopic measurements show enlargement of the outer diameter on all matrices. However, the data do not correlate with a decrease in average retention force losses. Thus, the increase in matrix diameter is greatest with the angled Novalocs (NY-15°), but is not reflected by a noticeably greater loss of retention force. The nonetheless higher wear verified in the marginal area of the yellow matrices may be due to the different shapes of the occlusal and lateral surfaces on the part of the manufacturer when compared to the straight Novaloc abutment. This is especially pertinent since microscopically, a tendency toward increased widening with greater implant angulation can be noticed (exceptions: NY-0° IA-20°; LP-0° IA-10°, Figure 8).

The adhesive effect influencing the retention force thus seems to be mainly characterized by the surfaces located in the outer undercut. This assumption is supported by the retention behavior of the pink Locator matrices. After ageing, the increased inner diameter of these (D2, Figure 8) did not stabilize the retention forces.

On the Novaloc attachment system, retention is also created by the open ring shape. The elasticity of the PEEK material permits bending over the bulge-like upper edge of the Novaloc abutment during adaptation of matrix and patrix. After complete joining, corresponding to the function of a denture clasp [45]—the ring lies flush again, due to the elastic recovery in the undercut area of the abutment.

### 4.5. Comparison of Release Periods During Retention Force Measurements

The release period was also influenced exclusively by the type of abutment and the respective matrix, but not by the implant angulation (Table 4). The straight polyamide abutments had the shortest release period (MWT: 0.00353 min), while the pink Locator matrices had significantly longer release periods (MWT: 0.0048 min). Compared to the literature, the periods determined were slightly shorter (Petropoulus 1997: 0.00547 min) [10]. Contrary to the information provided by Petropoulus, the release periods correlate with the retention forces (Table 2). However, the latter compared the release periods of fundamentally different attachments and can therefore not be used as a comparison. In conclusion, depending on the manual dexterity, the sometimes greatly increased retention forces of the pink Locators could have an effect on the implant in the form of horizontal shear forces during denture removal, even with greater force transmission [10]. However, a longer release period may reduce the risk of premature denture release when eating viscous food [18].

## 5. Conclusions

Within the limits of this study design, the following conclusions can be drawn:

1. The retention force specifications of the manufacturers are guide values and can vary considerably depending on the test parameters.
2. During the simulated wearing period of >9 years, all tested attachment systems showed wear reactions. With a retention force loss of 26.14%, the Novaloc attachment system showed a comparatively continuous curve. In contrast, the total retention force loss of the Locator attachment system of ~77.5% was in the clinically relevant range.
3. In the current study, implant angulation had no clinically relevant influence on the retention forces of the Novaloc system. With to the angled 15°—Novaloc abutment, divergences could be compensated, and identical retention forces could be determined in relation to the pull-off direction and abutment axis. Similarly, with the orange Locator inserts, no difference could be verified at varying implant angulations.
4. Future in vivo studies have to be conducted to verify the tendencies found in the current investigation. The angulation of implant abutments seems to be promising in terms of constant retention forces. Thus, it might be interesting to compare the clinical longevity of angulated attachment systems (e.g., Novaloc system) with currently introduced attachment systems that consist of matrices with a tilting mechanism (e.g., OT Equator system).

## Figures and Tables

**Table 1:** Materials and labeling of test series.

Attachment System (Manufacturer)	Description	Material	Labeling	Test Series–IA ^1^
Novaloc Retentive System (Institut Straumann AG, Basel, Switzerland)	RN Novaloc Abutment; 0° Gingiva height: 3 mm	Titanium grade 5 Amorphous Diamond-Like Carbon (ADLC)	N-0°	NY-0° IA 0° NY-0° IA 5° NY-0° IA 10° NY-0° IA 15° NY-0° IA 20°
	RN Novaloc Abutment; 15° Gingiva height: 3 mm	Titanium grade 5 Amorphous Diamond -Like Carbon (ADLC)	N-15°	NY-15° IA 15° NY-15° IA 20°
	Novaloc ^2^ retention Inserts yellow	Polyether ether ketone (PEEK)	Y	
Locator Attachment System (Zest Dental Solutions, Carlsbad, CA, USA)	RN Locator Abutment 0°-straight; Gingiva height: 3 mm	Titanium-aluminum-vanadium alloy (TAV)	L-0°	
	Locator ^3^ Replacement Male pink; light retention; angulation: 0°–10°; height: 1.7 mm	Nylon	P	LP-0° IA 0° LP-0° IA 5° LP-0° IA 10°
	Locator ^3^ Replacement Male orange; light retention; extended range; angulation: 10°–20°; height: 1.7 mm	Nylon	O	LO-0° IA 15° LO-0° IA 20°

^1^ IA: implant angulation; ^2^ fixed in matrix housing with attachment option, titanium; ^3^ fixed in denture caps (5.5 mm, height 2.5 mm).

**Table 2:** Mean values and standard deviation of retention force measurements with respect to the matrix color coding.

Matrix, Retention Inserts	Manufacturer Specifications	Retention Force Measurements					
		Initial		After Artificial Ageing (10,000 Cycles)		After Thermocycling	
(LP) Locator pink	3 Ibs (13.34 N) 1360 g	Mean 19.39 N ± 8.10 N	Median 17.8 N	Mean 6.46 N ± 2.62 N	Median 6.99 N	Mean 26.08 N ± 4.96 N	Median 25.92 N
(LO) Locator orange	2 Ibs (8.90 N) 910 g	Mean 8.8 N ± 5.28 N	Median 6.57 N	Mean 0.96 N ± 1.03 N	Median 0.66 N	Mean 11.77 N ± 4.16 N	Median 12.39 N
(NY) Novaloc yellow	2.7 Ibs (12.00 N) ca. 1200 g	Mean 8.57 N ± 0.99	Median 8.62 N	Mean 6.33 N ± 0.74 N	Median 6.34 N	Mean 5.72 N ± 0.63 N	Median 5.78 N

**Table 3:** Comparisons showing *p*-values ≤ 0.05 within a test series and ageing cycles; for labeling, refer to Table 1.

Series	A: Initial	B: 100 Cycles	C: 200 Cycles	D: 500 Cycles	E: 1000 Cycles	F: 5000 Cycles	G: 10,000 Cycles
*p*-value from	a) to	b) to	c) to	d) to	e) to	f) to	g) to
Retention NY-0° IA-0°	**8.71 N** f) 0.026 g) 0.025	**7.95 N**	**7.88 N**	**7.95 N**	**7.79 N**	**7.17 N** a) 0.026	**7.06 N** a) 0.025
Retention NY-0° IA-5°	**9.25 N** b, d, e) <0.001 c, f, g) 0.001	**8.46 N** a) <0.001 f) 0.016 g) 0.012	**8.50 N** a) 0.001 f) 0.010 g) 0.004	**8.32 N** a) <0.001 f) 0.015 g) 0.007	**8.16 N** a) <0.001 g) 0.015	**7.39 N** a) 0.001 b) 0.016 c) 0.010 d) 0.015 e) 0.015	**6.73 N** a) 0.001 b) 0.012 c) 0.004 d) 0.007
Retention NY-0° IA-10° ^1^	**8.10 N**	**7.80 N**	**7.43 N**	**7.59 N** g) 0.038	**7.62 N** g) 0.021	**6.90 N**	**6.48 N** d) 0.038 e) 0.021
Retention NY-0° IA-15°	**8.75 N** e) 0.006 f) 0.003 g) <0.001	**8.65 N** f) 0.001 g) <0.001	**8.27 N** g) <0.001	**8.24 N** g) <0.001	**8.14 N** a) 0.006 g) <0.001	**6.92 N** a) 0.003 b) 0.001	**5.95 N** a–e) <0.001
Retention NY-0° IA-20°	**8.21 N** f) 0.004 g) 0.015	**7.72 N** f) 0.005	**7.67 N** f) 0.011 g) 0.045	**7.86 N** f) 0.020 g) 0.022	**7.94 N** f) <0.001 g) 0.004	**6.59 N** a) 0.004 b) 0.005 c) 0.011 d) 0.020 e) <0.001	**5.89 N** a) 0.015 d) 0.022 e) 0.004
Retention NY-15° IA-15°	**8.60 N** b) 0.002 c) 0.044 f) 0.017 g) <0.001	**8.00 N** a) 0.002 g) 0.003	**8.05 N** a) 0.044 g) 0.001	**8.19 N** g) 0.001	**8.35 N** f) 0.017 g) 0.001	**7.22 N** a,e) 0.017 g) 0.008	**6.15 N** a) <0.001 b) 0.003 c–e) 0.001 f) 0.008
Retention NY-15° IA-20°	**8.34 N** g) 0.003	**8.21 N** g) 0.001	**8.07 N** g) 0.004	**8.08 N** e) 0.029 g) 0.001	**7.68 N** d) 0.029 g) 0.002	**7.70 N** g) 0.013	**6.07 N** a) 0.003 b, d) 0.001 c) 0.004 e) 0.002 f) 0.013
Retention LP-0° IA-0° ^1^	**25.41 N** f) 0.005 g) 0.006	**29.33 N** f) 0.019 g) 0.021	**29.26 N** e) 0.004 f, g) <0.001	**26.42 N** e) 0.002 f, g) <0.001	**15.78 N** c) 0.004 d) 0.002 f) 0.006 g) 0.005	**7.71 N** a) 0.005 b) 0.019 c, d) <0.001 e) 0.006	**7.83 N** a) 0.006 b) 0.021 c, d) <0.001 e) 0.005
Retention LP-0° IA-5° ^1^	**17.94 N** g) 0.04	**14.43 N** g) 0.039	**10.46 N** d) 0.002	**22.30 N** c) 0.002 e) 0.003 f, g) <0.001	**12.74 N** d) 0.003 f) 0.024 g) 0.002	**6.71 N** d) <0.001 e) 0.024	**4.42 N** a) 0.049 b) 0.039 d) <0.001 e) 0.002
Retention LP-0° IA-10° ^1^	**14.81 N** g) 0.014	**13.17 N** e) 0.010 g) 0.002	**10.51 N** d) 0.049 e) 0.001 g) 0.011	**22.06 N** c) 0.049 f) 0.027 g) 0.010	**20.62 N** b) 0.010 c) 0.001 f, g) <0.001	**9.41 N** d) 0.027 e) <0.001	**7.11 N** a) 0.014 b) 0.002 c) 0.011 d) 0.010 e) <0.001
Retention LO-0° IA-15° ^1^	**7.76 N** f) 0.045 g) 0.021	**4.63 N** f) 0.006 g) 0.001	**4.87 N** f) 0.003 g) 0.001	**8.59 N** f) 0.004 g) 0.002	**4.93 N** f) 0.009 g) 0.001	**2.29 N** a) 0.045 b) 0.006 c) 0.003 d) 0.004	**1.52 N** a) 0.021 b, c, e) 0.001 d) 0.002
Retention LO-0° IA-20° ^1^	**9.98 N**	**7.31 N**	**4.73 N** f) 0.043 g) 0.001	**4.91 N** f) 0.045 g) 0.001	**4.32 N** f) 0.041 g) <0.001	**2.15 N** c) 0.043 d) 0.045 e) 0.041	**0.39 N** c, d) 0.001 e) <0.001

^1^ Welch ANOVA, Levene’s test *p* ≤ 0.05.

**Table 4:** Release periods in ascending order.

Series ^1^	Release Period (min)
NY-0°–IA-0°	0.00314
NY-0°–IA-20°	0.00318
NY-0°–IA-15°	0.00355
NY-0°–IA-10°	0.00367
LO-0°–IA-15°	0.00368
LO-0°–IA-20°	0.00369
NY-15°–IA-20°	0.00377
NY-0°–IA-5 °	0.00413
NY-15°–IA-15°	0.00417
LP-0°–IA-5°	0.00459
LP-0°–IA-10°	0.00490
LP-0°–IA-0°	0.00492

^1^ Labeling see Table 1.
